# Towards a social functional account of laughter: Acoustic features convey reward, affiliation, and dominance

**DOI:** 10.1371/journal.pone.0183811

**Published:** 2017-08-29

**Authors:** Adrienne Wood, Jared Martin, Paula Niedenthal

**Affiliations:** Department of Psychology, University of Wisconsin – Madison, Madison, Wisconsin, United States of America; University of Sussex, UNITED KINGDOM

## Abstract

Recent work has identified the physical features of smiles that accomplish three tasks fundamental to human social living: rewarding behavior, establishing and managing affiliative bonds, and negotiating social status. The current work extends the social functional account to laughter. Participants (N = 762) rated the degree to which reward, affiliation, or dominance (between-subjects) was conveyed by 400 laughter samples acquired from a commercial sound effects website. Inclusion of a fourth rating dimension, spontaneity, allowed us to situate the current approach in the context of existing laughter research, which emphasizes the distinction between spontaneous and volitional laughter. We used 11 acoustic properties extracted from the laugh samples to predict participants’ ratings. Actor sex moderated, and sometimes even reversed, the relation between acoustics and participants’ judgments. Spontaneous laughter appears to serve the reward function in the current framework, as similar acoustic properties guided perceiver judgments of spontaneity and reward: reduced voicing and increased pitch, increased duration for female actors, and increased pitch slope, center of gravity, first formant, and noisiness for male actors. Affiliation ratings diverged from reward in their sex-dependent relationship to intensity and, for females, reduced pitch range and raised second formant. Dominance displayed the most distinct pattern of acoustic predictors, including increased pitch range, reduced second formant in females, and decreased pitch variability in males. We relate the current findings to existing findings on laughter and human and non-human vocalizations, concluding laughter can signal much more that felt or faked amusement.

## Introduction

The evil cackle of a B-movie villain. The polite smile of a stranger on a bus. The giggles, chuckles, and smile flashes that pepper conversation, making social interactions smooth and harmonious. These are recognizable instances of smiles and laughter, yet they defy the commonplace notion that “true” smiles mean people are happy and “true” laughter means people are amused [e.g.,^1^]. They suggest that smiles and laughter can do more than convey positive affect with varying degrees of authenticity [2,3,4].

Recent social-functional frameworks hold that facial expressions have evolved in the service of solving distinct problems posed by social living [5,6,7]. Thus, rather than asking whether an expression occurs during play versus competition, or whether it reflects a true or dissimulated emotion, a social functional approach asks how an expression influences the interrelated behavior of the expresser and observer(s) in the service of meeting social opportunities and challenges (see also [8]). As applied to a particularly complex expression, the human smile, one social-functional theory holds that three basic tasks of social living—*rewarding the self and others*, *maintaining social bonds*, and *negotiating social hierarchies*—can be accomplished by the encoding of specific displays called reward, affiliation, and dominance smiles, respectively. Supporting empirical work has identified the unique morphological features of the three smiles [9]. Further, validation studies have confirmed that the expressions are spontaneously categorized as smiles, and convey the distinct meanings of reward, affiliation, and dominance [10].

Smiles frequently co-occur with laughter [11], and thus might constitute different aspects of a single over-arching social gesture [12,13]. Given the primacy of vocalizations in the communication of many mammalian species, smiles may have gained specific social meanings due to their influences on physical properties of the voice [14,15,16,17,18]. In particular, smiling shortens the vocal tract, increasing the space between the first and second formants [19]. Changes in formant positioning capture the attention of rhesus macaques [20], suggesting it conveys important social information. Indeed, increased spacing between formants creates the illusion of a smaller body size [21,22]. Across mammalian species, vocalizations that make the producer seem smaller and/or less dominant, such as rapid, high-pitched vocalizations, are used in social contexts to signal appeasement, playfulness, fear, and other non-threatening or pro-social intentions [23,24,25]. When human ancestors evolved voiced as opposed to unvoiced laughter, it would have been adaptive to pair laughter with smiling, as the air expulsion during spontaneous laughter cannot be easily modulated [3]. We propose that lip positioning during laughter bouts can be more readily modulated to increase or decrease the small body illusion. Different smiles could therefore modulate the signal carried by laughter and other vocalizations.

In light of the likely structural relationships between smiles and laughter, the present work links the function of the two signals. We propose that, like smiles, laughter serves to solve problems of reward, affiliation, and social status negotiation, and we explore the acoustic properties that are linked to the perception of these meanings.

### A social functional account of laughter

There is evidence that humans laugh in a variety of ways in order to influence their social worlds [26] and convey a range of intentional states [27, 28, 29]. Thus, the extension of a social-functional analysis of smiles to laughter seems theoretically feasible. However, to date, empirical basis for this extension is lacking. Extant research focuses largely on physical properties of laughter that predict perceptions of spontaneity, or the degree to which a laugh sounds uncontrollable versus intentional [e.g., 3, 30], with spontaneous laughter associated with greater positivity and friendliness [12, 31].

Spontaneous laughs originate from evolutionarily ancient subcortical brain regions, involve spasms of the diaphragm, override motor commands from higher brain regions (such that it is difficult to speak during spontaneous laughter), and have unique acoustic attributes [28]. In social functional terms, spontaneous laughter may be experienced as rewarding. Thus, like spontaneous laughter, laughs that act as reward signals are probably especially salient [32], contagious [33,34], and enjoyable for producers [35] and listeners [36]. An affiliation laugh in the present framework most likely corresponds to the previously-identified “social” laughter, which often occurs in non-humorous social encounters and appears to serve relationship maintenance [25,28] and conversation smoothing [27] functions. Like its smile counterpart, laughter of affiliation in theory serves to efficiently indicate that the subject of the laughter intends no threat and that the relationship itself is not in danger.

Evidence suggests that laughter can also signal aggressive intentions [37,38,39]. Conveying dominance and superiority with laughter may be more effective than with more overtly negative and aggressive behavior that invites conflict (for theorizing on ritualized threat displays, see [40]). Laughing *at* someone and their inferiority could be the ultimate signal of the laugher’s superior status, signaling the laugher is so far above the target in status that they do not need to engage in direct conflict to prove it. Somewhat separately, laughter that conveys dominance could be useful for enforcing norms within a group [41].

Szameitat and colleagues [29] demonstrated the perceptual discriminability of laughs produced by actors imagining themselves in different contexts/feeling states, including being tickled, taunting someone, feeling joy, and feeling schadenfreude (pleasure at another’s misfortune). The posed schadenfreude and taunting laughter, which fit into the current framework’s conceptualization of dominance laughter, differed acoustically from the posed joy and tickle-induced laughter and was perceived more negatively by raters. Laughs that signal affiliation and dominance intentions would most likely be categorized as “voluntary” or lacking spontaneity [3]. However, this does not mean that laughter that communicates information other than positivity and reward is consciously-controlled or faked—they are likely automatized and honest signals of social intentions [42].

Further evidence that laughter might serve the social functions of reward, affiliation, and dominance comes from research on humor. Humor is often accompanied by and is intended to elicit laughter [43]. Humor can be used and enjoyed purely for the pleasurable feelings it produces [44]. But it can also serve the function of ingratiation and connecting with a group [45] or specifically to signal one’s higher status to listeners [46]. Taken together, the evidence suggests that laughter is used to serve the same basic tasks as smiles and that these two gestures are part of a similar social signaling system.

### Overview of the present work

The present exploratory research was designed to extend a social functional account of smiles [6] to laughter. We presented 400 laughter samples to participants in an online study. In a between-subjects design, we asked participants to rate the degree to which each laugh clip expressed meanings related to reward, affiliation, dominance, or spontaneity. We included the latter in order to compare the current framework to the primary diagnostic dimension in the literature, but we do not consider spontaneity to be a fourth candidate social function. Spontaneous laughs are defined by their neural and physiological underpinnings [3], while social functions are identified by their behavioral outcomes. Thus participants’ judgments about how spontaneous a laugh seems could be orthogonal to or correlated with their social functional judgments.

We chose not to use laughter samples obtained in a lab setting or from a naturalistic database, as both options will always be limited by the social contexts the researchers chose to record. Instead, we used laughter bursts from a professional online sound library (soundsnap.com), with the assumption that a resource for videogame and movie sound editors would include vocalizations meant to convey a wide range of social intentions. We extracted relevant acoustic variables from the laugh samples and used them to predict subjects’ social function ratings in a series of linear mixed-effect models with actor sex as a moderator. Each of the social functional dimensions was associated with a distinct acoustic profile, and spontaneity and reward were largely overlapping. Many of the social judgments related to different acoustic properties for male and female vocalizations.

## Method

This study was conducted according to the appropriate ethical guidelines and approved by the Institutional Review Board (IRB) at the University of Wisconsin—Madison. Participants were at least 18 years old and were fully informed of what the study involved. Because obtaining signed consent was impractical in the online study, the IRB approved a waiver for signed consent. No sensitive information was collected, and all data were confidential. We analyzed only anonymous data. We report all data exclusions, all manipulations, and all measures. The data and analysis files and all laughter clips are available online (https://osf.io/ca66s/).

### Participants and procedure

We recruited 768 online participants on Amazon’s Mechanical Turk and TurkPrime [47] to “rate 50 very brief audio clips of people laughing” in exchange for $2 (all participation occurred May 11–12, 2017). Five participants reported audio malfunctions and one participant reported that he did not listen to the sounds before rating them; excluding these participants resulted in a sample of 762 (see Table 1 for participant demographics).

**Table 1:** Participant demographics.

	**n**	**%**
**Gender**		
Female	328	43.04
Male	430	56.43
Other	2	0.26
(not reported)	2	0.26
**Race/Ethnicity**		
American Indian or Alaska Native	6	0.79
Asian	62	8.14
Black or African American	56	7.35
Native Hawaiian or other Pacific Islander	2	0.26
Non-Hispanic White	563	73.88
Hispanic or Latino/a	48	6.3
Other or mixed ethnicity	25	3.28
	**M (SD)**	**Min-Max**
**Age (years)**	37.72 (10.77)	21–77

After reading the consent information, participants were randomly assigned to judge the degree to which the laugh samples communicated a meaning related to one of the four dimensions (spontaneity *n* = 172, reward *n* = 254, affiliation *n* = 166, dominance *n* = 170). Each participant evaluated the laughs on just one of the four rating scales so experimental demands would not lead them to rate each laugh as high on only one dimension. Due to a programming error, the reward condition was oversampled.

Each participant rated a subsample of 50 laughs randomly drawn from the entire pool of 400 laughs. Each laugh was rated on a given dimension approximately 24 times (762 participants *50 judgments) / (400 laughs * 4 rating dimensions). Instructions asked participants to rely on their “spontaneous impressions” to “rate the extent to which you think the…description fits this clip”. The descriptions, which varied across conditions, were accompanied by a 10-point Likert scale (1 = “not at all”, 10 = “very much”):

- **Spontaneity condition**: “Laughter can sometimes be spontaneous. You could feel that someone’s laughter is unintentional and is occurring outside of their control.”
- **Reward condition:** “Laughter can sometimes be rewarding. You could feel that someone’s laughter means they like something that you did or said.”
- **Affiliation condition:** “Laughter can sometimes be reassuring. You could feel that someone’s laughter means they are acknowledging you and want you to know they are not threatening.”
- **Dominance condition:** “Laughter can sometimes be mocking. You could feel that someone’s laughter means at this moment they feel superior to or dominant over you.”

After rating 50 laughs, participants answered several demographic and task feedback questions.

### Laughter stimuli

To maximize the variability of our laughter sample, we obtained our stimuli from Sound Snap, a professional online sound library (soundsnap.com). Sound Snap’s voice recordings are licensed by sound designers and producers; as such, they are largely produced in recording studios and often sound artificial. This is particularly important to consider in laughter, as spontaneity strongly influences perceiver judgments. However, we think it is appropriate to use these somewhat artificial stimuli in the current study for two reasons. Firstly, our social functional account is agnostic about the feeling states underlying an expression, instead seeking to identify common social consequences. Secondly, posed and synthetic facial expressions have been instrumental in identifying the action units relevant to certain emotions or social functions [48], and distilled, sometimes caricatured expressions often exaggerate the most essential features of an expression [49].

On April 19, 2017, we used the following keywords in a Sound Snap search, which returned 598 audio clips: LAUGH* -*BOY* -*GIRL* -CARTOON* -GROUP* -CROWD* -ANIMAL -WOMEN -MEN -LADIES -KID -BAB* -TODDLER* -TALK* -SPEECH -SPEAK* -MANIC (dashes precede excluded keywords). Clips were then eliminated from the initial search return for the following reasons: contained no adult human laughter; contained speech, ambient noise, or multiple speakers; were low-quality vintage recordings; or were tagged with the words “ghost,” “clown,” “cartoon,” or “crazy.” This resulted in 400 relevant laughter samples (256 male, 144 female). We then trimmed any silence from the beginning and end of the samples.

### Acoustic feature extraction

Eleven acoustic features were extracted from the 400 laugh samples using PRAAT [50] (see Table 2 for descriptive statistics). We describe the variables and the motivation for their inclusion in the current study below:

- **Duration:** The *duration* of the laughter sample in seconds, log-transformed to correct for positive skew. In at least one study, spontaneous laughter bouts were longer than volitional bouts [30, cf 3].
- **Intensity:** The mean *intensity*, or loudness, in dB. Greater intensity may be an indicator of reduced inhibition [51] or increased laughter spontaneity [3].
- **Pitch variables:** *F0 mean* refers to mean fundamental frequency, or pitch, as calculated using PRAAT’s auto-correlation technique. *F0 range* is the difference between the lowest and highest F0 for each sample. *Standard deviation of F0 divided by the total duration* (*SD F0 / duration*) of the sample captures the average moment-to-moment variability in pitch; this variable was log-transformed to correct for positive skew. *Slope* is the mean absolute F0 slope, which measures how sharply the pitch changes occur by dividing the difference between a local F0 maximum and minimum (at intervals of .01 seconds) by the duration it takes to go from one to the other. Raised F0 and greater SD F0 / duration and F0 range are associated with spontaneity in laughter [3,30]. Steeper F0 slopes are associated with high arousal emotion states [52]. To correct for the skewed distribution of pitch variables on a Hertz scale, F0 mean, slope, and F0 range were transformed from Hertz to a semitone scale (12*log(X)), with F0 range calculated as a ratio of the maximum to minimum F0 (12*log(maximum/minimum)) [3].
- **Spectral variables:** *Center of gravity* refers to the spectral centroid, which accounts for the weighting of noise across the sample (log-transformed). Changes in center of gravity can correspond to the oral-nasal distinction in vowels [53] and the perception of vowel height in nasal vowels [54]. More generally, center of gravity is an indicator of the timbre, or brightness, of a sound, with higher centers sounding brighter [55]. Spontaneous laughs in one study had higher centers of gravity than volitional laughs [30]. *Harmonics-to-noise ratio* is the average degree of periodicity in dB; a higher value indicates a purer, more tonal sound, and a lower value indicates a noisier vocalization. *Proportion voiced* is the proportion of frames that are voiced as opposed to unvoiced. Voiced segments are nearly periodic, while unvoiced segments are noisier, and include exhalations and snorts [12]. Previous work showed that spontaneous laughs have more unvoiced segments [30] and longer intervals between voiced bursts [3] compared to volitional laughs. Laughs intended to portray teasing and schadenfreude have lower harmonics-to-noise ratios than laughter intended to portray tickling [29]. The spectral variables should be interpreted cautiously due to the laughter samples’ unknown recording environments and possible compression at some point in the editing process.
- **Formant variables:** *F1 mean* and *F2 mean*, or the first and second formants (transformed to semitones), are peaks in the sound spectrum that help determine the vowel sound of a vocalization. Lowering F1 and raising F2 results in a “higher” vowel (e.g., shifting from /aː/ to /iː/). Spontaneous and rewarding laughter may be expected to feature high F1 means based on previous research [56], as a higher F1 is associated with higher arousal [57]. Raised F2 can convey increased positivity [58]. A general raising of the vowel sound, which involves increasing the relative dispersion of the first and second formants, creates the illusion of a smaller body size [19], as formant spacing is much more strongly related to body size than F0 [59,60]. Furthermore, open vowel sounds are associated with high-arousal calls in monkeys [61]. Formant positioning therefore has the potential to predict perceptions of all four social dimensions in laughter [21].

**Table 2:** Summary statistics for the 11 acoustic measures and subjective ratings for the 395 laughs included in the key analyses, separated by the sex of the actor (female *n* = 142, male *n* = 253).

		Mean	SD	Median	Min	Max	Skew	Kurtosis
Duration (s)*	Females	2.71	3.01	1.46	.11	17.53	2.30	6.45
	Males	2.24	2.51	1.39	.11	16.05	2.87	10.06
**Intensity (dB)**	Females	71.95	5.14	72.38	58.83	84.47	-.29	-.07
	Males	66.92	8.46	68.18	36.71	83.51	-.81	.65
**F0 mean (Hz)**	Females	411.44	129.00	394.80	194.56	855.87	0.81	0.65
	Males	313.18	133.92	290.76	89.07	794.16	0.93	0.77
**F0 mean (ST)**	Females	71.67	3.71	71.74	63.25	81.03	-.04	-.20
	Males	67.89	5.14	68.07	53.87	80.13	-.17	-.17
**F0 Range (ST)**	Females	13.27	7.71	12.20	1.65	31.25	.56	-.58
	Males	16.00	8.13	16.87	.23	32.30	-.13	-1.11
SD F0/Duration*	Females	83.26	87.53	51.80	5.43	498.21	2.28	6.15
	Males	106.58	166.24	61.08	2.77	1778.72	6.49	55.27
**Slope (ST)**	Females	82.64	6.77	82.75	62.53	99.79	-.26	.38
	Males	80.96	10.2	80.99	49.05	105.12	-.36	-.21
Center of Gravity*	Females	1097.65	497.49	1049.76	93.5	2416.48	.46	-.37
	Males	983.51	417.24	932.23	236.64	2527.66	.79	1.00
**Harmonics-to-Noise Ratio**	Females	8.81	3.89	8.50	.69	2.60	.49	.06
	Males	7.48	4.48	6.16	.23	26.83	1.36	2.11
**Proportion Voiced**	Females	.49	.22	.48	.04	1.00	.17	-.68
	Males	.47	.23	.45	.05	1.00	.40	-.49
**F1 Mean (ST)**	Females	81.03	3.05	81.63	69.64	87.51	-.85	.97
	Males	80.64	3.11	80.97	65.66	86.71	-1.19	3.07
**F2 Mean (ST)**	Females	90.27	1.26	90.26	86.06	95.00	.07	1.36
	Males	89.53	1.81	89.55	84.63	94.87	-.12	.01
**Spontaneity**	Females	4.53	2.70	4	1	10	.25	-1.09
	Males	5.13	3.04	5	1	10	.09	-1.29
**Reward**	Females	4.89	2.75	5	1	10	.15	-1.11
	Males	4.47	2.85	4	1	10	.30	-1.17
**Affiliation**	Females	5.53	2.66	6	1	10	-.16	-1.00
	Males	4.77	3.00	5	1	10	.25	-1.24
**Dominance**	Females	5.83	2.60	6	1	10	-.19	-.93
	Males	5.27	2.78	6	1	10	-.03	-1.14

*Note*. Where indicated, pitch variables have been transformed to a semitone scale (ST) for analyses, although F0 Mean is also reported in Hertz. F0 Range is the change in semitones from the minimum to the maximum pitch.

*Indicates that a variable was subsequently log-transformed for analyses due to non-normality, identified via visual inspection.

Five laugh samples were removed from subsequent analyses because they had no voiced frames and were therefore missing values for pitch variables. Inspecting the summary statistics suggests participants rated these unvoiced laughs as lower on reward (*M* = 2.84, *SD* = 2.23), affiliation (*M* = 3.04, *SD* = 2.29), and dominance (*M* = 4.26, *SD* = 3.23) than the other 395 laughs (mean spontaneity ratings are not noticeably different, *M* = 4.82, *SD* = 3.05, see Table 2 for descriptives of included laughs).

## Results and discussion

### Analytic strategy

**Fig 1:**
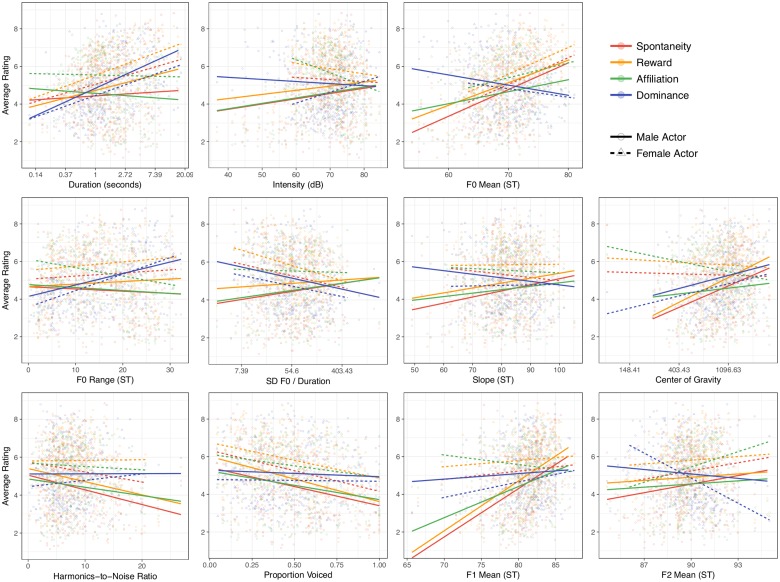
The relationship between laugh samples’ acoustic measures and average participant ratings, separated by actor sex, fitted with ordinary least squares regression. *Footnotes*. Each point is a single laugh sample’s average score for a given social judgment. Y axes are the degree to which participants thought the description of each social dimension fit the laugh (1 = not at all, 10 = very much). X-axis tick marks for log-transformed variables are non-linear because they have been converted back to the original unit of measure. ST indicates a pitch variable that has been converted to the semitone scale. Since the plotted regression lines are from simple regressions, they may not perfectly match the coefficients from the reported LMEMs in Table 4.

We conducted a series of linear mixed-effect models (LMEM) to identify which acoustic variables predict variability in the social functional ratings. Separately for each social judgment dimension * acoustic variable combination (see Table 3 for correlations between the dimensions), we regressed participants’ raw responses on the acoustic variable (see Fig 1 for scatterplots for each variable). In all models, we included interactions between the acoustic variable and actor sex, given the sex differences in acoustic properties of laughter [62], frequency of “social” laughter [27], and the social acceptability of dominance displays [63].

**Table 3:** Pearson’s correlation coefficients for the judgment dimensions, based on each laugh’s average rating (female actors / male actors).

	Spontaneity	Reward	Affiliation	Dominance
Spontaneity	-			
Reward	.84 / .85	-		
Affiliation	.60 / .72	.71 / .77	-	
Dominance	-.43 / -.47	-.41 / -.33	-.72 / -.62	-

All *p*’s < .001. Female df = 140, male df = 251.

Analyses were conducted in the R environment [64] using the lme4 package [65] for model fitting and the lmerTest package [66] for calculating denominator degrees of freedom using Satterthwaite’s approximations. In each of these models, we regressed participants’ raw responses (on a scale from 1–10) on interactions between actor sex and one of each of the following acoustic variables (plus the two lower-order terms): log-transformed duration, intensity, F0 mean, F0 range, log-transformed F0 SD/ duration, slope, log-transformed center of gravity, harmonics-to-noise ratio, proportion voiced, F1 mean, and F2 mean. Since multiple observations were made for each laugh sample, we included a by-laugh random intercept. We included by-subject random intercept and random slopes for actor sex and the acoustic variable, since they vary within-subject. In four cases where we encountered model convergence failures, we constrained the covariance between random effects to zero [67]. Because we estimated 44 unique models (4 social judgment dimensions X 11 acoustic variables), we controlled the false discovery rate by reporting Benjamini-Hochberg adjusted p values, which were computed separately for each of the four outcome measures [68]. See supplementary materials for the results of an alternate analytic strategy in which we simultaneously regressed all 11 acoustic variables onto each social dimension, resulting in just 4 models but less interpretable estimates.

**Fig 2:**
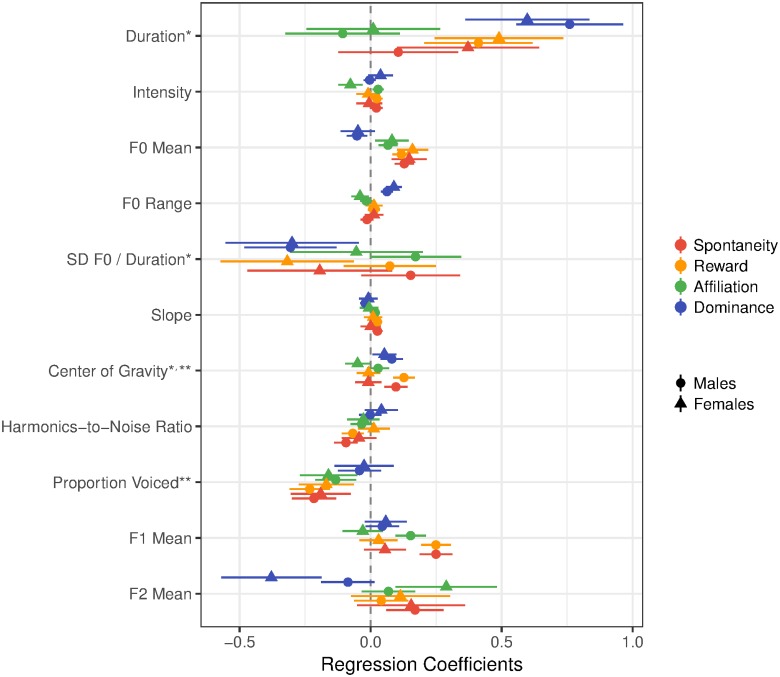
Unstandardized regression coefficients and 95% confidence intervals for models predicting spontaneity, reward, affiliation, and dominance ratings for male and female actors. *Footnotes*. Estimates from models in which the sex variable was centered over males (circles) and when it was centered over females (triangles), illustrating the effects of the acoustic variables on social judgments separately for male and female actors. Since unstandardized regression coefficients are reported, comparisons are best made within each variable rather than across variables, as the scales are different. *Indicates coefficient and standard error for a log-transformed predictor. **Indicates the coefficient and standard error were divided by 10 to better match the size of the other coefficients.

The initial models used centered actor sex (male = -.5, female = .5), but were followed up by models in which actor sex was recoded so that either male or female was coded as zero; this allowed us to identify which acoustic variables predict social judgments specifically for male and female actors. In the following sections we summarize and interpret the significant predictors for each social judgment outcome, but see Table 4 and Fig 2 for complete model estimates.

**Table 4:** Model estimates from LMEMs predicting social judgments from acoustic variables and their interactions with actor sex.

	Spontaneity Models				Reward Models				Affiliation Models				Dominance Models			
Variable	b	SE	t	adj. p	b	SE	t	adj. p	b	SE	t	adj. p	b	SE	t	adj. p
Duration*	.238	.095	2.501	.029	.451	.086	5.215	<.001	-.048	.090	-.536	.686	.679	.085	8.013	<.001
Sex Interaction	.266	.170	1.565	.226	.079	.154	.510	.672	.116	.161	.725	.591	-.162	.148	-1.091	.391
Males	.105	.116	.905	.473	.411	.106	3.894	<.001	-.107	.111	-.959	.480	.760	.104	7.314	<.001
Females	.371	.138	2.687	.022	.490	.125	3.916	<.001	.010	.130	.075	.940	.598	.121	4.957	<.001
**Intensity**	.008	.014	.560	.704	.007	.013	.531	.672	-.024	.013	-1.813	.141	.017	.013	1.256	.318
Sex Interaction	-.028	.027	-1.022	.410	-.033	.024	-1.369	.294	-.106	.025	-4.194	<.001	.042	.026	1.592	.229
Males	.022	.012	1.806	.150	.024	.011	2.093	.077	.028	.011	2.571	.034	-.004	.012	-.334	.813
Females	-.006	.025	-.233	.876	-.010	.023	-.432	.714	-.077	.024	-3.275	.006	.038	.024	1.581	.229
**F0 Mean**	.138	.020	6.855	<.001	.139	.018	7.560	<.001	.074	.020	3.738	.002	-.050	.020	-2.519	.036
Sex Interaction	.019	.037	.512	.724	.042	.033	1.267	.335	.015	.036	.422	.733	.004	.037	.097	.967
Males	.128	.019	6.853	<.001	.117	.018	6.678	<.001	.066	.019	3.572	.003	-.052	.020	-2.673	.024
Females	.147	.034	4.352	<.001	.160	.030	5.267	<.001	.081	.033	2.486	.038	-.049	.033	-1.462	.264
**F0 Range**	-.001	.011	-.051	.982	.013	.010	1.197	.362	-.029	.011	-2.740	.023	.076	.010	7.572	<.001
Sex Interaction	.026	.021	1.253	.320	.000	.019	.008	.994	-.024	.019	-1.213	.341	.026	.018	1.431	.264
Males	-.014	.012	-1.104	.372	.012	.012	1.057	.427	-.017	.012	-1.468	.242	.062	.011	5.486	<.001
Females	.013	.018	.710	.601	.013	.016	.773	.541	-.041	.017	-2.450	.038	.089	.015	5.742	<.001
SD F0 / Duration*	-.021	.087	-.238	.876	-.122	.081	-1.504	.255	.058	.081	.723	.591	-.303	.080	-3.798	.001
Sex Interaction	-.347	.167	-2.080	.084	-.391	.153	-2.565	.026	-.226	.153	-1.480	.242	.006	.156	.041	.968
Males	.153	.096	1.594	.223	.073	.090	.816	.540	.171	.089	1.922	.122	-.306	.090	-3.416	.003
Females	-.194	.141	-1.377	.298	-.318	.130	-2.452	.034	-.055	.129	-.424	.733	-.300	.130	-2.312	.058
**F0 Slope**	.013	.011	1.169	.345	.017	.010	1.697	.181	.006	.010	.539	.686	-.015	.010	-1.431	.264
Sex Interaction	-.026	.021	-1.221	.326	-.016	.019	-.829	.540	-.024	.020	-1.195	.341	.012	.020	.589	.679
Males	.026	.010	2.631	.023	.025	.009	2.739	.017	.017	.009	1.886	.126	-.021	.009	-2.200	.069
Females	.000	.019	-.004	.997	.009	.018	.528	.672	-.006	.018	-.347	.764	-.009	.018	-.486	.746
Center of Gravity*	.437	.173	2.527	.029	.593	.162	3.669	.001	-.107	.171	-.625	.651	.666	.161	4.125	<.001
Sex Interaction	-1.053	.328	-3.208	.005	-1.351	.297	-4.557	<.001	-.783	.306	-2.557	.034	-.286	.313	-.913	.455
Males	.964	.224	4.302	<.001	1.269	.208	6.099	<.001	.285	.215	1.329	.298	.809	.216	3.745	.001
Females	-.089	.252	-.355	.815	-.082	.230	-.357	.738	-.498	.243	-2.050	.097	.523	.233	2.240	.066
**Harmonics-to-Noise Ratio**	-.070	.021	-3.314	.004	-.028	.019	-1.447	.272	-.031	.019	-1.625	.192	.020	.020	1.003	.422
Sex Interaction	.052	.039	1.311	.304	.080	.036	2.190	.064	.006	.037	.175	.881	.042	.039	1.103	.391
Males	-.094	.023	-4.138	<.001	-.068	.021	-3.217	.004	-.034	.021	-1.639	.192	-.001	.022	-.068	.968
Females	-.044	.034	-1.303	.304	.012	.031	.382	.736	-.028	.031	-.891	.514	.041	.032	1.272	.318
**Proportion Voiced**	-2.036	.374	-5.447	<.001	-2.007	.340	-5.909	<.001	-1.472	.349	-4.217	<.001	-.339	.366	-.927	.455
Sex Interaction	.256	.702	.364	.815	.633	.643	.984	.462	-.275	.674	-.408	.733	.176	.689	.255	.857
Males	-2.164	.431	-5.023	<.001	-2.324	.392	-5.928	<.001	-1.335	.399	-3.343	.006	-.427	.416	-1.027	.419
Females	-1.908	.583	-3.273	.004	-1.691	.533	-3.172	.004	-1.609	.558	-2.885	.016	-.251	.576	-.436	.768
**F1 Mean**	.152	.026	5.788	<.001	.140	.024	5.748	<.001	.061	.025	2.477	.038	.054	.026	2.061	.092
Sex Interaction	-.194	.051	-3.800	.001	-.216	.046	-4.685	<.001	-.183	.049	-3.759	.002	.020	.052	.383	.792
Males	.249	.032	7.825	<.001	.249	.029	8.654	<.001	.153	.030	5.159	<.001	.044	.032	1.363	.283
Females	.055	.041	1.346	.303	.030	.037	.812	.540	-.030	.039	-.764	.591	.058	.041	1.422	.264
**F2 Mean**	.162	.061	2.669	.022	.077	.057	1.362	.294	.178	.057	3.109	.010	-.233	.056	-4.130	<.001
Sex Interaction	-.015	.116	-.128	.941	.073	.106	.687	.585	.221	.108	2.038	.097	-.292	.108	-2.706	.024
Males	.170	.056	3.048	.008	.041	.053	.768	.541	.068	.052	1.313	.298	-.087	.052	-1.668	.212
Females	.155	.105	1.476	.258	.114	.096	1.180	.362	.289	.099	2.923	.016	-.379	.097	-3.891	.001

The first rows for each variable are the model estimates for the main effects (averaged across male and female actors). The second rows are the estimates for the acoustic variable-by-sex interaction terms. The third and fourth rows are the effects of the acoustic variables when the sex term is recoded, so they indicate the effect for males and females, respectively. P values are Benjamini-Hochberg adjusted for multiple comparisons. Significant effects are in color: significant main effects are green, interaction terms are orange, simple effects for male actors are yellow, and simple effects for females are blue.

*Indicates a log-transformed variable.

### Acoustic features associated with spontaneity and reward

We found judgments of spontaneity and reward to be highly correlated (*r* = .84) and predicted by many of the same acoustic properties (see Table 4, “Spontaneity Models” and “Reward Models”). Increased perceptions of spontaneity and reward were associated with higher F0 means, a feature likely influenced by arousal levels of the expresser and that is observed in research on spontaneous laughter [3,30]. Also replicating previous work [30], laughs high on perceived spontaneity and reward had less voicing.

In addition to having higher F0 means and less voicing, female but not male laughter was perceived as more spontaneous when it was longer in duration [see also 30]. This is perhaps due to the fact that females are normatively expected to be, and are, less intrusive with speech and vocalizations [69]. When a female does laugh longer, it may seem to perceivers that the laughter is truly outside of her control, while perceivers may not expect male volitional laughter to be constrained. In contrast to spontaneity, perceptions of reward were predicted by laugh sample duration for both males and females. If future work replicates this pattern, it supports the notion that males are generally less inhibited in their laughter, so variability in male laughter bout length is informative about the social function of a laugh, but not informative of how uncontrollable the laughter is.

We see further sex-specific effects for spontaneity. Compared to females, judgments of male spontaneity were predicted by a more complex pattern: increased F0 slope, higher spectral center of gravity, increased F1 and F2 means, and reduced harmonics-to-noise ratio. Previous work has linked reduced harmonics-to-noise ratio to perceptions of positivity, and increased center of gravity to perceptions of arousal [30]. Increased F0 slope [50] and raised F1 means [55] have been associated with high-arousal emotional states. F2 means are positively related to perceivers’ judgments that a vocalization reflects an intense emotion [55] or a positive affective state [57], and higher F2 creates a higher vowel sound. At least in the current stimulus set, judgments of males’—but less so for females’—spontaneity appear to have been guided by biologically-reliable indicators of arousal and valence.

Sex-dependent effects on reward judgments differed from spontaneity for two acoustic variables in addition to bout duration: pitch variability and F2 mean. SD F0 / duration (i.e. pitch variability) was negatively associated with perceptions of female reward. Pitch variability was not predictive of spontaneity judgments here or in previous work [30], although that same study showed that spontaneously-elicited laughter actually features *greater* pitch variability than volitional laughter, a feature perceivers did not seem to pick up on. Future work should determine whether this relationship between pitch variability and perceived reward of female laughter is an artifact of the current study or a feature that distinguishes rewarding functions from outright spontaneity. The male-specific relationship between F2 mean and spontaneity was absent for reward.

### Acoustic features associated with affiliation

Perceptions of affiliation, like spontaneity and reward, were associated with higher pitch and reduced voicing for males and females, and a male-specific effect of F1 mean. The remaining acoustic predictors of affiliation judgments were unique to affiliation and sex-specific (see Table 4, “Affiliation Models”).

Affiliation was the only social judgment predicted by the intensity, or loudness, of a laugh, and exhibited opposite patterns for males and females. Males were judged as conveying appeasement and non-threatening intentions when their laughter was louder, while female laughter sounded more affiliative when it was quieter. If females are expected to be generally more restrained [70], then they might be perceived as friendlier and less threatening with quieter laughter, while outgoing-sounding, loud laughter might sound more acceptable and friendlier in males. Indeed, disinhibition is an attractive quality in males [71]. This is speculation and requires follow-up research.

F0 range, or the distance in semitones between the minimum and maximum pitch of a laugh bout, was negatively associated with affiliation judgments for female actors. Threat and high-arousal states in non-human primates are conveyed with large jumps in pitch, while low-arousal vocalizations involve smaller pitch changes [59]. The interaction between sex and spectral center of gravity in the affiliation model was significant, with a larger, more negative simple effect for females, but this female-specific effect was not significant after correcting for multiple comparisons. A lower center of gravity conveys lower arousal and more volitional laughter [30], and might therefore signal non-threatening, soothing intentions.

Further distinguishing affiliation from reward and spontaneity, female laughter with higher F2 means was perceived as more affiliative. Higher second formants occur in positive affective vocalizations in humans [57] and are perceived as a signal of smaller body size, conveying appeasement and submission in animals [59]. Raising F2 produces higher-sounding vowels and can be accentuated with retracted lips: for instance, compared to neutral lips, retracted lips shift the vowel /yː/ up to /iː/ [19]. This suggests a possible relationship between the degree to which a laugh “sounds” like a smile and, at least in females, how affiliative it sounds.

### Acoustic features associated with dominance

The only features shared between laughs perceived as highly dominant and laughs perceived as spontaneous/rewarding are longer durations and higher centers of gravity for male actors. Dominance and affiliation are not predicted by any of the same acoustic features, and relate to several acoustic variables in opposite directions (see Table 4, “Dominance Models”). See Table 5 for a summary of properties shared by the social dimensions.

**Table 5:** Summary of distinct and shared acoustic predictors of the four social judgments.

	**Spontaneity**	**Reward**	**Affiliation**	**Dominance**
**Spontaneity**	↑ Duration (F)	↑ Duration (F*)		↑ Duration (F*)
	↑ F0 Mean	↑ F0 Mean	↑ F0 Mean	
	↑ Slope (M)	↑ Slope (M)		
	↑ Center of Gravity (M)	↑ Center of Gravity (M)		↑ Center of Gravity (M)
	↓ Harmonics-to-Noise Ratio (M)	↓ Harmonics-to-Noise Ratio (M)		
	↓ Proportion Voiced	↓ Proportion Voiced	↓ Proportion Voiced	
	↑ F1 Mean (M)	↑ F1 Mean (M)	↑ F1 Mean (M)	
	↑ F2 Mean (M)			
**Reward**	↑ Duration (F*)	↑ Duration		↑ Duration
	↑ F0 Mean	↑ F0 Mean	↑ F0 Mean	
		↓ SD F0 / Duration (F)		
	↑ Slope (M)	↑ Slope (M)		
	↑ Center of Gravity (M)	↑ Center of Gravity (M)		↑ Center of Gravity (M)
	↓ Harmonics-to-Noise Ratio (M)	↓ Harmonics-to-Noise Ratio (M)		
	↓ Proportion Voiced	↓ Proportion Voiced	↓ Proportion Voiced	
	↑ F1 Mean (M)	↑ F1 Mean (M)	↑ F1 Mean (M)	
**Affiliation**			Intensity (M↑,F↓)	
	↑ F0 Mean	↑ F0 Mean	↑ F0 Mean	
			↓ F0 Range (F)	
	↓ Proportion Voiced	↓ Proportion Voiced	↓ Proportion Voiced	
	↑ F1 Mean (M)	↑ F1 Mean (M)	↑ F1 Mean (M)	
			↑ F2 Mean (F)	
**Dominance**	↑ Duration (F*)	↑ Duration		↑ Duration
				↓ F0 Mean (M)
				↑ F0 Range
				↓ SD F0 / Duration (M)
	↑ Center of Gravity (M)	↑ Center of Gravity (M)		↑ Center of Gravity (M)
				↓ F2 Mean (F)

Each cell contains the acoustic predictors that were significant and in the same direction for both the row and column social dimension; the diagonal contains all significant predictors for a given social dimension. Variable names followed by an (M) or (F) had significant effects for only one sex.

*Indicates a shared effect that predicts one of the social dimensions for both males and females, but predicts the other social dimension for only one sex.

Green cells and arrows pointing up (↑) indicate a positive regression coefficient, and red cells and arrows pointing down (↓) indicate a negative coefficient; yellow indicates the relationship between the acoustic property and the social dimension is the opposite for males and females.

Lower F0 means and SD F0 / duration both predict perceptions of dominance, but these main effects appear to be driven by male actors. These are properties shared with non-laugh vocalizations that convey dominance and largeness in humans and non-human animals [23, 72,73,74]. In an unsurprising reversal of F2’s relationship to perceptions of affiliation in females, female laughs with lower F2 means are perceived as more dominant (the interaction term here is significant, suggesting no effect of F2 on perceptions of male dominance).

More dominant laughs have greater F0 ranges for both males and females. This relationship is unexpected as spontaneous laughter tends to have a greater pitch range than volitional laughter [3,30], and given the divergence of dominance and spontaneity ratings, we expected dominant laughter to have a lower range. This puzzle may be clarified in future work examining the pitch contour of a laughter bout: laughter conveying dominance and superiority may have a strong downward pitch contour with little variability, like more “dominant” speech utterances (e.g., statements as opposed to questions, [75]).

Another surprising (non-)effect on dominance perceptions is harmonics-to-noise ratio. Animal threat vocalizations are typically noisier [59], and posed laughter intended to portray *schadenfreude* and taunting has been observed to be noisier than laughter portraying tickling and joy [29]. We should avoid drawing conclusions from this null result, but future work should explore if and when noisiness is a reliable predictor of perceived dominance.

### Possible explanations of observed sex differences

There are several potential explanations for why actor sex moderates the relationships between acoustic properties and social judgments. The first possibility is that spontaneous, rewarding, affiliative, and dominant laughs sound different when produced by males versus females [60]. The human vocal apparatus is sexually dimorphic [76] and male and female actors modulate different acoustic features to portray laughter in various social contexts [29]. The second possibility is that, specifically in our laugh sample set, the male and female actors conveyed different social intentions in distinct and, possibly, stereotypical ways, so that if we reproduced the current study using naturalistic laughter, the sex differences would disappear. For instance, it could be that males sometimes convey affiliation in ways similar to females, but this was just not represented in the Sound Snap database. The final possibility is that the sex differences are due to participants’ mental models of how males and females sound when they are being spontaneous, rewarding, affiliative, and dominant. In line with this, previous work suggests that male listeners disregard acoustic cues of female laughter spontaneity [77]. Regardless of the source of these sex differences, this work highlights sex as an important moderator of social signals, particularly for behaviors like laughter with sexually dimorphic physiology, and when studying highly gendered social tasks like affiliation and dominance.

### Possessing versus expressing affiliation and dominance

We must reconcile the acoustic signatures of affiliative laughter here with previous work, which shows that listeners can detect affiliation in dyads [78]. This work showed that listeners from many different cultures can detect when co-laughter is between friends or strangers, with laughter between friends involving acoustic features associated with spontaneity. Why, then, does the present work suggest separable acoustic signatures of spontaneity and affiliation? The previous study operationalized “affiliation” as whether co-laughers have an established relationship, while here we operationalize signals of affiliation as cues of appeasement and non-threat. It is sensible that laughter produced in the presence of a friend is more spontaneous (and therefore rewarding), and such laughter reinforces close bonds [79]. Affiliative laughter as we define it is a tool for signaling friendliness and benign intentions, and such signals are only necessary when those intentions cannot be inferred or taken for granted. Spontaneous and rewarding co-laughter may indicate a secure social bond, while affiliative co-laughter may indicate bond maintenance or establishment is occurring. Indeed, such a distinction occurs in the facial displays of mandrill monkeys [80].

A similar clarification must be made for dominance. A recent study examined the acoustic properties of laughs emitted by group members possessing different levels of actual power and status as they jokingly teased each other [49]. This study revealed a strikingly different pattern of results than those presented here: laughter produced by dominant group members had higher pitch, pitch variability, and intensity, among other outcomes. The dominant laughers, overall, seem to be producing more spontaneous and disinhibited laughs compared to the low-status laughers. As with affiliation, this confusion can be reconciled by realizing that the previous study defined “dominant” individuals as those possessing actual power and status, while we are focused on signals intended to *exert* dominance on others [for a similar distinction in pride displays, see 81]. Such signals are hypothesized to occur when people perceive a discrepancy between their actual and deserved status in the group, or as a way to discount another person’s status.

### Limitations of the current work

The nature of the current study’s laugh samples is a limitation that future research should remedy. First, we were unable to control for possible non-independence due to the same voice actors producing multiple laugh samples. Besides being statistically problematic, an inability to group samples by actor prevented us from using acoustic variables like nasality, which is best analyzed within-actor [82]. Future work should include additional relevant acoustic variables, including intervoicing interval [3], pitch contours [24], and apparent vocal tract length [21].

Another limitation of the sample used is that they were largely posed (albeit by presumably professional voice actors), so the spontaneity dimension was restricted. One could make the conservative assumption that none of the 400 laugh samples are spontaneous in an underlying neural and physiological sense, but still, many of the acoustic features predicting *perceptions* of spontaneity in the current work match those previously observed. In our social functional approach, which is agnostic about the underlying internal state or physiology of the expresser, it is arguably most important that the current study’s stimuli had variability in perceptions of spontaneity. Still, future work should explore whether, with verifiably spontaneous laughter, perceptions of reward and spontaneity continue to be based on overlapping acoustic features.

### Summary

We predicted participants’ ratings for each of the social judgment dimensions—spontaneity, reward, affiliation, and dominance—using 11 acoustic variables in order to identify the systematic acoustic properties listeners use to extract social meaning from laughter. Similar acoustic features guided perceptions of spontaneity and reward, with just a few exceptions, and some of these acoustic features have been previously identified as diagnostic of spontaneity [3,30]. We therefore suggest spontaneous laughs can serve a rewarding function. Affiliation judgments shared a few characteristics with spontaneity and reward, but several acoustic features distinctly predicted affiliation. Dominance judgments related to the most distinct pattern of acoustic features, often relating to the acoustic variables in direction opposite to the other social judgment dimensions.

Interestingly, actor sex was an important moderator of the relationship between many acoustic properties and perceivers’ judgments. Besides the reliable indicators of spontaneity—proportion voiced and F0 mean—female spontaneity and reward judgments were based on a sparser set of predictors (duration and variability, with the latter only predicting reward). A more complex set of variables predict judgments of male spontaneity and reward.

Female laughter that conveys affiliation involves acoustic properties associated with signals of appeasement and friendliness [19,57], such as raised pitch, raised second formant, and reduced intensity. The pattern of acoustics that convey affiliation in males, meanwhile, bears some resemblance to higher-arousal, more spontaneous states (e.g., greater intensity combined with higher pitch and first formant) [3,55]. Beyond the signatures of dominant intentions shared by males and females, the communication of dominance in males involves lower pitch and higher spectral center of gravity, while in females it involves a lowered vowel (as reflected by F2).

This study is an exploratory first step towards a social functional account of laughter. It complements the spontaneity distinction, which by itself is insufficient to predict what form laughter will take across a variety of social contexts [83,84,85,86]. Because laughter conveys that any accompanying behaviors are harmless, it allows expressers to act upon their social worlds without risking relationships [87].

We hope this initial step towards applying the same social functional approach to both smiles and laughter will inspire future research to integrate across expressive modalities. Future research will test whether laughter produced in vivo does indeed serve the social tasks of reward, affiliation, and dominance, and will explore if and how different variations of laughter co-occur with different variations in smiles [9]. The social functional approach generates useful predictions about the adaptive origins and social consequences of nonverbal expressions, going beyond more traditional approaches that focus on the affective or physiological state of the expresser.
